# Temporal depolarization of mitochondria during M phase

**DOI:** 10.1038/s41598-017-15907-3

**Published:** 2017-11-22

**Authors:** Kotoe Hirusaki, Kaho Yokoyama, Kyunghak Cho, Yoshihiro Ohta

**Affiliations:** Division of Biotechnology and Life Sciences, Institute of Engineering, Tokyo University of Agriculture and Technology, Nakacho 2-24-16, Koganei, Tokyo, 184-8588 Japan

## Abstract

Mitochondrial activity in cells must be tightly controlled in response to changes in intracellular circumstances. Despite drastic changes in intracellular conditions and mitochondrial morphology, it is not clear how mitochondrial activity is controlled during M phase of the cell cycle. Here, we show that mitochondrial activity is drastically changed during M phase. Mitochondrial membrane potential changed during M phase progression. Mitochondria were polarized until metaphase to the same extent as mitochondria in interphase cells, but were depolarized at around telophase and cytokinesis. After cytokinesis, mitochondrial membrane potential was recovered. In addition, the generation of superoxide anions in mitochondria was significantly reduced at metaphase even in the presence of antimycin A, an inhibitor of complex III. These results suggest that the electron supply to the mitochondrial electron transfer chain is suppressed during M phase. This suppression might decrease the reactive oxygen species generated by the fragmentation of mitochondria during M phase.

## Introduction

Mitochondria play important roles in cellular metabolism; they provide most ATP required by cells and are linked to many metabolic systems. In addition, reactive oxygen species (ROS) are mainly produced by the electron transfer chain (ETC) in mitochondria^1–3^. High ROS generation results in oxidative stress, leading to cellular damage, but ROS act as signal molecules at sublethal concentrations^4–6^.

To maintain the important roles of mitochondria, mitochondrial activities are finely tuned in response to changes in intracellular circumstances^7–9^. Changes in intracellular conditions during cell cycle progression are associated with metabolic and morphological changes of mitochondria^10^. In the G1 phase, mitochondria are fragmented^11^, mitochondrial functions are inhibited^12^, and glycolysis is highly activated^13^. The G1/S transition is regulated by mitochondria via ROS and AMP^14^ as well as fragmentation^11^. At the G1/S transition, mitochondria are converted from isolated and fragmented elements to a giant hyperfused network and have greater ATP output than that of mitochondria at any other cell cycle stage^11^. In the G2/M phase, ATP production is highly dependent on mitochondrial respiration^13^, and complex I in ETC is activated by cyclin B1/Cdk1^15^. In the early M phase, mitochondria are fragmented^16–18^. The inhibition of mitochondrial fragmentation results in the arrest of cells at G2/M interphase^17^. The fragmented mitochondria form a tubular structure after telophase^19^.

Intracellular circumstances change drastically during M phase. Cyclin- dependent kinases (Cdks) are inactivated after metaphase^20^, and this might explain changes in mitochondrial activity. Despite extensive studies of morphological changes, changes in mitochondrial activity during M phase are not well-understood. Here, we show that the supply of electrons to the ETC in mitochondria is transiently decreased, especially after metaphase. These results suggest that both ATP and ROS generation by mitochondria are transiently suppressed during M phase.

## Results

### Fluorescence analysis of cells undergoing morphological changes

**Figure 1:**
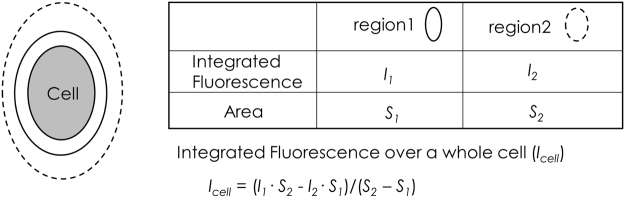
Schematic illustration of the integrated fluorescence analysis. Grey area shows a cell. Region 1 (solid ellipse) encompasses a whole cell. Region 2 (broken ellipse) encompasses region 1. Neither region (1 or 2) contains cells or fluorescent obstacles other than the cell of interest. *I*_cell_ is the integrated fluorescence over a whole cell. *I*_1_ and *I*_2_ are the integrated fluorescence values within region 1 and region 2, respectively. *S*_1_ and *S*_2_ are the areas of region 1 and region 2, respectively.

**Figure 2:**
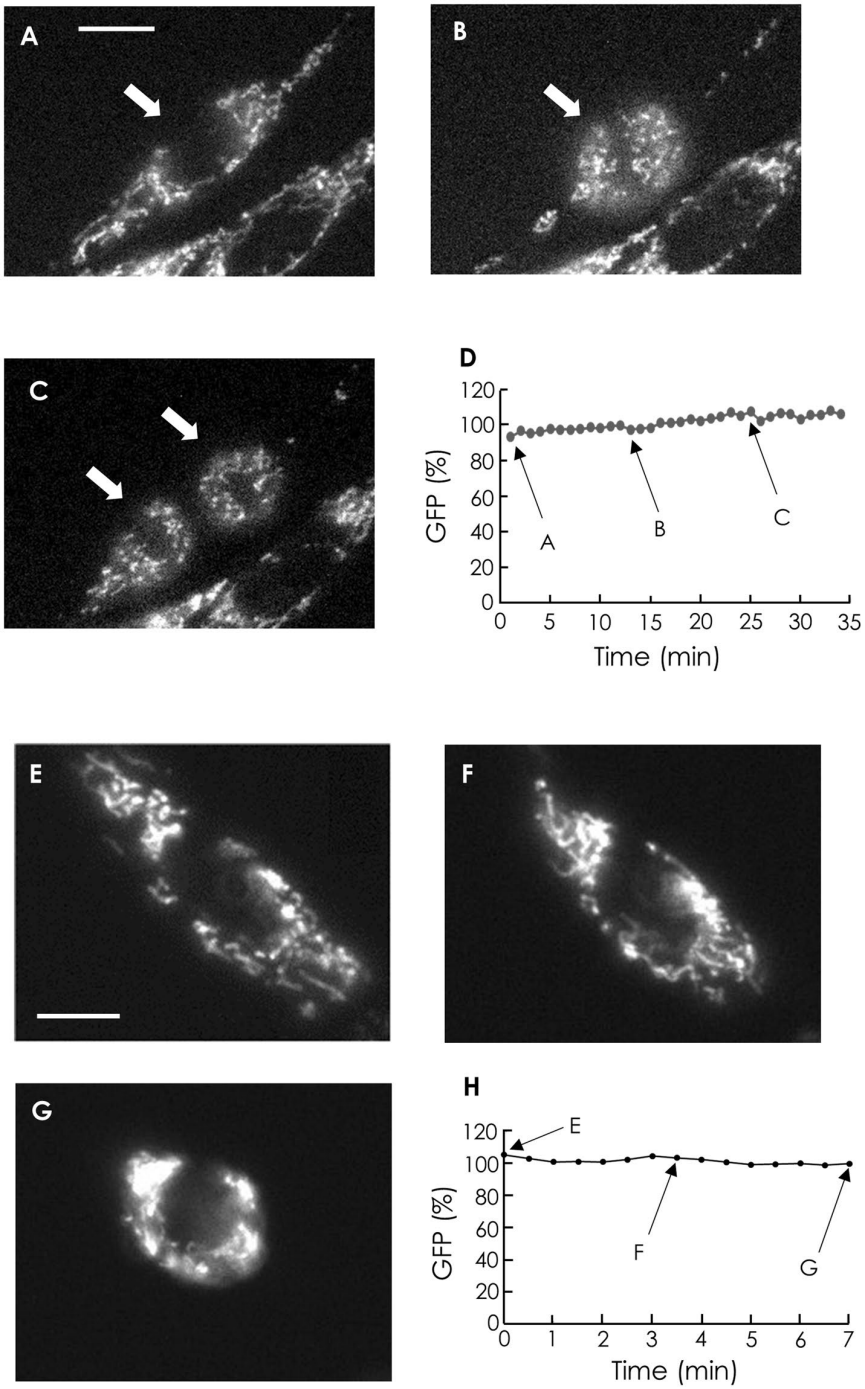
Stability of integrated fluorescence during morphological changes in cells. (**A**–**C**) Fluorescence images of GFP expressed in mitochondria in C6 cells during cell division. Bar, 10 μm. (**D**) Typical time course of the integrated fluorescence of GFP in a single cell during division. Arrows A–C show the integrated fluorescence intensities over a whole cell, indicated by arrows in (**A**), (**B**), and (**C**), respectively. The fluorescence intensity at t = 5 is normalized to 100. In the case of C, the fluorescence intensity is the sum of the integrated fluorescence of two daughter cells. (**E**–**G**) Fluorescence images of GFP expressed in mitochondria in C6 cells during cell shrinkage. Shrinking was induced by the addition of 0.05% trypsin in HBS without Ca^2+^ and bovine serum albumin. Bar, 10 μm. (**H**) Typical time course of integrated fluorescence of GFP in a single cell during cell shrinkage. Arrows E–G show the integrated fluorescence of a cell indicated in (**E**), (**F**), and (**G**), respectively. The fluorescence intensity at t = 1 is normalized to 100.

During M phase, the morphologies of cells and mitochondria change drastically^21^. Since morphological changes are expected to affect the average fluorescence intensity in a certain area within a cell, we measured the integrated fluorescence intensity over a whole cell, instead of the average fluorescence intensity, as the fluorescence intensity independent of cell morphology (Fig. 1). To confirm that integrated fluorescence is not significantly affected by cell morphology, we expressed GFP in mitochondria of C6 cells and evaluated integrated fluorescence during M phase (Fig. 2A–C). The integrated fluorescence intensity did not differ among stages during M phase, as shown in Fig. 2D. In contrast, the average fluorescence in cells changed substantially during M phase. For further confirmation, we induced morphological changes in cells at interphase by treatment with trypsin (Fig. 2E–G). Similar to the fluorescence intensity results obtained during M phase, integrated fluorescence remained unchanged during cell rounding induced by trypsin (Fig. 2H). The integrated fluorescence of tetramethylrhodamine ethyl ester (TMRE), a mitochondrial membrane potential-sensitive dye, was also unchanged during cell rounding induced by trypsin. These results indicate that integrated fluorescence over a cell can be used as a measure of cell fluorescence, independent of the cell morphology.

### Changes in mitochondrial membrane potential during cell division

**Figure 3:**
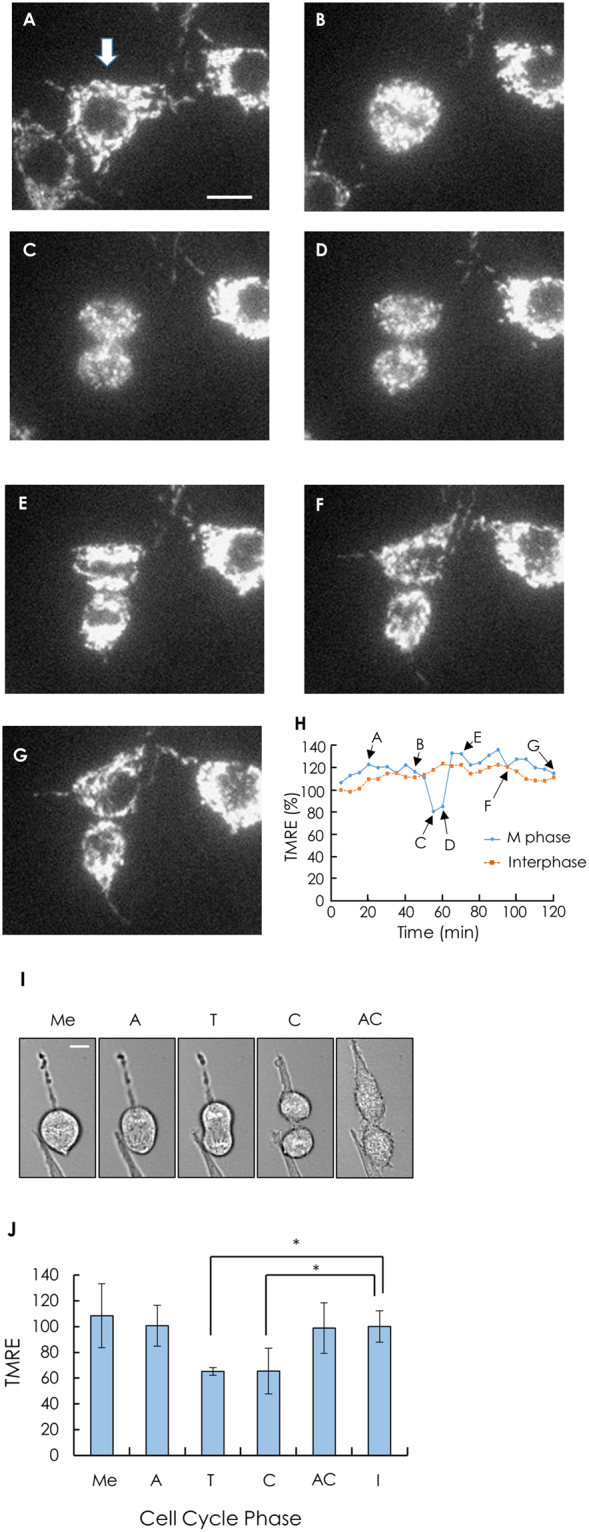
Changes in mitochondrial membrane potential during cell division. (**A**–**G**) TMRE fluorescence images of a cell during cell division. Bar, 10 μm. (**H**) The integrated fluorescence of TMRE during cell division. The integrated fluorescence over a whole cell was measured every 5 min. The blue line shows TMRE fluorescence changes in a cell in M phase (a cell indicated by an arrow in A). The red line shows those in a cell at interphase in the same microscopic field. The TMRE fluorescence in the interphase cell at t = 5 min is normalized to 100. (**I**) Morphology of a cell at each stage during cell division. Me, metaphase; A, anaphase; T, telophase; C, cytokinesis; AC, after cytokinesis. Bar, 10 μm. (**J**) TMRE fluorescence changes during M phase. The transmittance and TMRE fluorescence images of single cells were measured every minute. The stages of a single dividing cell were determined based on the transmittance images. Me, A, T, C, and AC indicate stages, as described above. I, Interphase. The average of the integrated fluorescence of TMRE in the interphase cells in the same microscopic field is normalised to 100. Data were analysed by repeated measure analysis of variance. Results were obtained from five independent experiments (N = 5). Data are presented as means ± S.E.M. *P < 0.05.

To observe mitochondrial activity during cell division, we measured the fluorescence of TMRE, a mitochondrial membrane potential-sensitive dye, in cells. During M phase, the cell with thread-like mitochondria (Fig. 3A) shrank, and the thread-like mitochondria were fragmented after metaphase (Fig. 3C and D). After cytokinesis, mitochondrial morphology recovered to the thread-like structure as the cell size increased again (Fig. 3F and G). Since morphological changes in cells could affect mitochondria via the consumption of ATP, we analysed the integrated TMRE fluorescence over a cell. As shown in Fig. 3H, TMRE fluorescence in a cell transiently decreased during M phase. Changes in TMRE fluorescence at interphase in the same microscopic field varied substantially among cells. To examine when mitochondria are most depolarized during M phase, we measured the integrated TMRE fluorescence in a dividing cell at 1-minute intervals. Fluorescence was lowest at telophase and cytokinesis (Fig. 3I and J). These results indicate that mitochondria gradually depolarize from metaphase to telophase, and then repolarize after cytokinesis to the level observed at interphase.

### Generation of ROS by mitochondria during M phase

**Figure 4:**
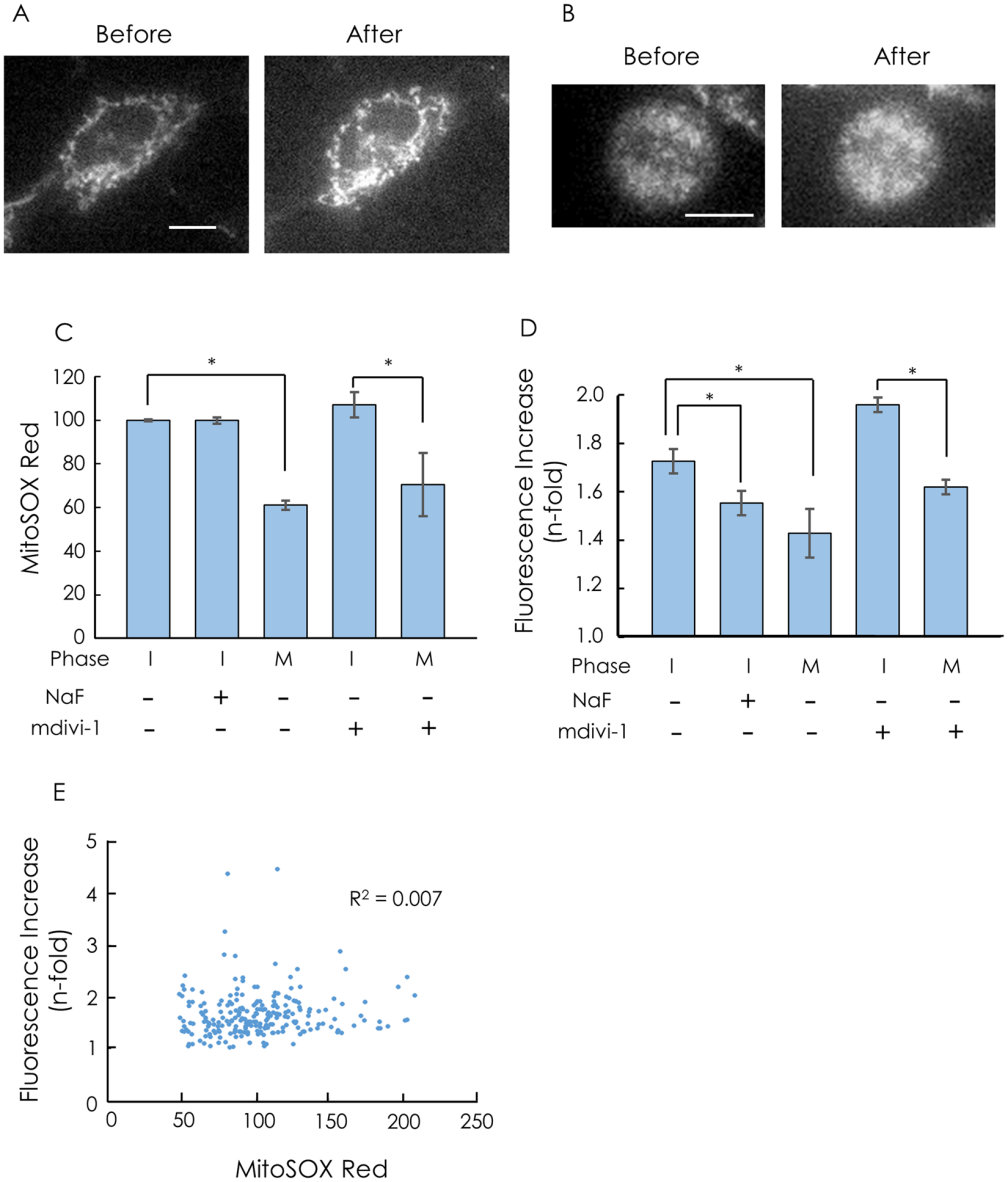
ROS production in cells during cell division. (**A**,**B**) Fluorescence images of MitoSOX Red in C6 cells before and after the addition of antimycin A. (**A**) interphase, (**B**) metaphase. Bar, 10 μm. (**C**) MitoSOX Red fluorescence in cells. The fluorescence was integrated over a whole cell. I, interphase; M, metaphase. The average of the integrated fluorescence of cells at interphase was normalized to 100. When glycolysis was inhibited, C6 cells were incubated with 5 mM NaF in HBS without glucose prior to staining with MitoSOX Red. Cells were incubated with 20 μM mdivi-1 for 6 h at 37 °C in the CO_2_ incubator prior to the measurements. Data are presented as means ± S.E.M. n = 13–54. *P < 0.05. (**D**) Increase in MitoSOX Red fluorescence by the addition of antimycin A. The increase is denoted as the ratio of the fluorescence 5 min after the addition of 1 μM antimycin A to the fluorescence before the addition of antimycin A. Data are expressed as means ± S.E.M. n = 13–54. *P < 0.05. (**E**) The correlation between MitoSOX Red fluorescence before the addition of antimycin A and the increase in MitoSOX Red fluorescence by the addition of antimycin A. MitoSOX Red fluorescence and the increase by the addition of antimycin A were measured for the same cells at interphase. n = 228. *R* ^2^, squared correlation coefficient.

Mitochondria were transiently depolarized during M phase. Next, we measured superoxide anion generation by mitochondria in cells during M phase with MitoSOX Red, a fluorescent indicator of superoxide anions^22^. Fluorescence images of MitoSOX Red are shown in Fig. 4A and B. The integrated fluorescence intensity of MitoSOX Red was significantly lower for metaphase cells than interphase cells (Fig. 4C). This result indicates that superoxide anion production by mitochondria was lower at metaphase than at interphase because there was no significant difference in mitochondrial membrane potential between cells at these phases (Fig. 3J) and because MitoSOX Red enters mitochondria in a membrane potential-dependent manner^23^.

Mitochondrial depolarization and the suppression of ROS generation could result from the suppression of the electron supply to the ETC; accordingly, we measured MitoSOX Red fluorescence in the presence of antimycin A, an inhibitor of complex III, to estimate the electron supply to the ETC in single dividing cells. This experiment is based on the idea that the amount of superoxide anion produced in mitochondria reflects the quantity of electrons supplied to the ETC when the ETC is inhibited, since a superoxide anion is produced by the leakage of an electron from the ETC^1–3^. The fluorescence intensity of MitoSOX Red increased in response to antimycin A (Fig. 4A and B). The increase in MitoSOX Red fluorescence, expressed as the ratio of MitoSOX Red fluorescence after the addition of antimycin A to that before the addition of antimycin A, was significantly suppressed in cells in which glycolysis was inhibited by NaF, an inhibitor of enolase (Supplementary Fig. S1, Fig. 4D). Pyruvate produced by glycolysis stimulates the supply of electrons to the ETC; accordingly, these results are consistent with the relationship between superoxide anion production and electrons supplied to the ETC. We also confirmed that there was no significant correlation between the increase in MitoSOX Red fluorescence and MitoSOX Red fluorescence before the addition of antimycin A in cells at interphase (Fig. 4E). Taken together, the increase in MitoSOX Red fluorescence by antimycin A reflects the amount of electrons supplied to the ETC, and does not depend on the fluorescence intensity before the addition of antimycin A.

The antimycin A-induced increase in MitoSOX Red fluorescence in cells was significantly smaller at metaphase than at interphase (Fig. 4D). This result is consistent with the idea that the electron supply to the ETC is significantly depressed in metaphase. In addition, mdivi-1, an inhibitor of mitochondrial fission^24^, did not affect the suppression of the electron supply to the ETC during M phase, although mdivi-1 decreased the percentage of fragmented mitochondria (Supplementary Fig. S2) and reduced the percentage of cells at metaphase that completed cell division from 100% to 55% This suggests that mitochondrial fission does not explain the depression of the electron supply to the ETC during M phase.

### Effects of antimycin A on cell damage

**Figure 5:**
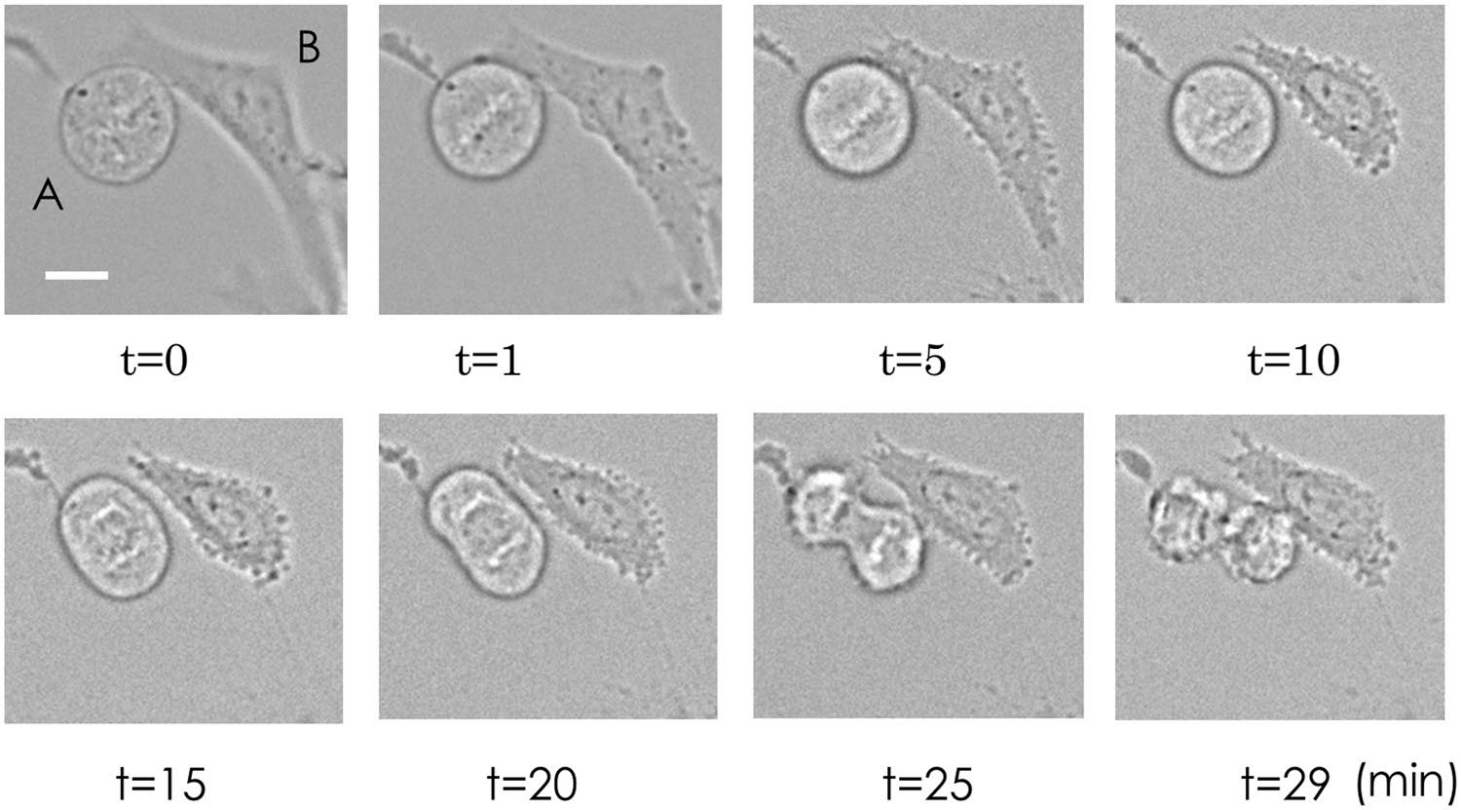
Antimycin A-induced damage during cell division. Transmittance images were obtained every min. At t = 0 min, antimycin (**A**) was added at 2 μM. A, a cell in M phase; (**B**), a cell in interphase. Bar, 10 μm.

During M phase, the supply of electrons to the ETC could be decreased. For further confirmation, we compared antimycin A–induced damage at metaphase and interphase. As shown in Fig. 5, interphase cells began to shrink and showed blebbing 5 min after the addition of antimycin A, indicating that antimycin A significantly and quickly damaged interphase cells. However, metaphase cells continued to undergo cell division and showed blebbing 25 min after the addition of antimycin A. Since cell damage induced by antimycin A results from the inhibition of electron transfer through the ETC, these results are consistent with the transient suppression of the electron supply to the ETC during M phase.

## Discussion

During cell division, mitochondria were transiently depolarized at around telophase. In addition, ROS production decreased, probably due to the decrease in electrons supplied to the ETC.

During M phase, mitochondria undergo fragmentation^16^. Since fragmentation is reported to induce mitochondrial depolarization^25–27^, the observed depolarization might be due to fragmentation, rather than a decrease in the supply of electrons to the ETC. However, we inferred that the decrease in the supply of electrons to the ETC better explains the observed depolarization because a decrease in ROS production was also observed in the presence of mdivi-1, which inhibits mitochondrial fission. Electrons are supplied to the ETC mainly via the oxidation of pyruvate and fatty acids. The observed decrease in the supply of electrons to the ETC is probably due to the suppression of electrons from both sources. This is supported by the observation that the suppression of glycolysis, leading to a decrease in pyruvate oxidation, did not induce the rapid depolarization of mitochondria in cells at interphase, as observed during M phase.

**Figure 6:**
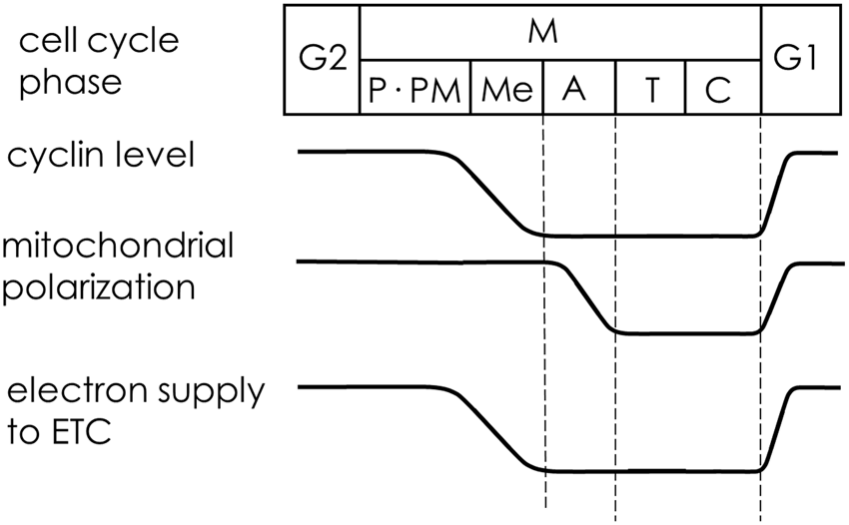
Hypothesis for changes in the mitochondrial energy state during cell division. P, prophase; PM, prometaphase; M. metaphase; A, anaphase; T, telophase: C, cytokinesis. The cyclin level shown refers to ref.^20^.

The mechanism underlying the decrease in the supply of electrons to the ETC is unclear. However, based on the present observations, we hypothesize that the inactivation of cdks decreases the supply of electrons to the ETC, resulting in the depolarization of mitochondria, and the re-activation of cdks recovers the electron supply and mitochondrial polarization (Fig. 6) because most cdks are inactivated for the metaphase to anaphase transition^20^. In fact, cyclin B1/cdk1 phosphorylates complex I and activates it at the G2/M transition^15^. Therefore, the inactivation of complex I due to dephosphorylation should result in a decrease in the amount of electrons supplied to the ETC. Since mitochondrial fragmentation occurs at the early stage of M phase^16^ and since fragmentation stimulates ROS generation^25–27^, if the quantity of electrons supplied to the ETC does not decrease during M phase, the fragmentation of mitochondria will be harmful to cells owing to enhanced ROS generation. Therefore, the observed decrease in the electron supply can protect cells by preventing the enhanced generation of ROS.

In the present study, we examined the cell cycle progression without trapping cells at a certain stage; this approach minimizes artefacts derived from cell trapping. For this reason, since the stage of the cell cycle depends substantially on the cell, we measured single cells instead of the cell ensemble by optical microscopy. Measurements of single cells also enabled us to determine the exact correspondence between changes in the mitochondrial membrane potential and the stages of the cell during division. In addition, we successfully detected the difference in electron supply to the ETC in a single cell. Although this is a semi-quantitative measurement, it will be useful for cell ensembles containing heterologous cells.

## Methods

### Reagents

C6 cells were obtained from the Institute of Fermentation (Osaka, Japan). The vector pAcGFP1-Mito was purchased from Clontech Laboratories (Mountain View, CA, USA). Tetramethylrhodamine ethyl ester (TMRE) and MitoSOX Red were purchased from Thermo Fisher Scientific (Waltham, MA, USA). All other high purity chemicals were commercially available.

### Cell Cultures

C6 cells were maintained in Dulbecco’s Modified Eagle’s Medium supplemented with 10% foetal bovine serum at 37 °C in a humidified atmosphere of 5% CO_2_. C6 cell lines expressing GFP in the mitochondria were obtained by plasmid transfection using Lipofectamine 2000 (Invitrogen, Carlsbad, CA, USA). For the expression of GFP, after 48 h of transfection, the cells were transferred to media containing 800 μg/ml geneticin^28^. Surviving cells were grown for 2–3 weeks, and colonies were picked. GFP expression in mitochondria was confirmed by fluorescence microscopy. These cells were cultured on glass-bottom culture dishes coated with polyethyleneimine for 2–3 days before microscopic observation.

### Lactate Determination

To evaluate lactate, cells were incubated for 15 min at 37 °C in DMEM without FBS. When NaF was added, the cells were pre-incubated for 10 min in the presence of 5 mM NaF, and NaF was present for the 15-min incubation period. After incubation, lactate in the medium was determined using a commercially available kit for L-lactate detection according to the manufacturer’s instruction (F-Kit; Roche, Basel, Switzerland). The protein content in cells was determined using the BCA protein assay with BSA as a standard.

### Fluorescence staining and imaging

To observe changes in the membrane potential of mitochondria, C6 cells were stained with 50 nM TMRE in culture medium for 10 min and observed in a humidified atmosphere of 5% CO_2_ at 37 °C. To obtain fluorescence images, an inverted epifluorescence microscope was used. The magnification and numerical aperture of the objective lens were 40 times and 0.9, respectively. TMRE fluorescence was monitored using a 75-W xenon lamp through a 15-nm bandpass filter centred at 535 nm. Fluorescence > 580 nm was collected using a cooled CCD camera^29,30^. To observe ROS production by mitochondria, C6 cells were stained with 2.5 μM MitoSOX Red in HEPES-buffered saline (HBS) (10 mM HEPES, 120 mM NaCl, 4 mM KCl, 0.5 mM MgSO_4_, 1 mM NaH_2_PO_4_, 4 mM NaHCO_3_, 1.2 mM CaCl_2_, 25 mM glucose, 0.1% bovine serum albumin, pH 7.4) for 10 min and observed at 37 °C. Fluorescence of MitoSOX Red was observed as described for TMRE. For GFP fluorescence, excitation at 480 nm and emission at 532 nm were collected. All images were obtained with an exposure time of 1 s. The fluorescence readouts were digitized to 12 bits and analysed using image-processing software.

### Analysis of Integrated Fluorescence

Since cells exhibit drastic morphological changes during cell division, the integrated fluorescence intensity of cells was measured, instead of the average fluorescence intensity within a region of interest, as shown in Fig. 1. The integrated fluorescence of a cell (*I*_cell_) was calculated according to equation (1),

1 $${I}_{cell}=({I}_{1}\cdot {S}_{2}-{I}_{2}\cdot {S}_{1})/({S}_{2}-{S}_{1})$$

where *I*_1_ and *S*_1_ are the integrated fluorescence intensity and area for a small region surrounding the cell, respectively, and *I*_2_ and *S*_2_ are the integrated fluorescence intensity and area for a large region surrounding the small region (see Fig. 1). We chose the regions 1 & 2 to include only the whole cells of interest and the background. The smaller region (region 1) should be sufficiently large to contain the pixels near the cells that show higher values than the pixels in the background.

### Statistical analysis

Data are expressed as means ± S.E.M. ANOVA followed by the Student-Newman-Keuls test was used to analyse differences among groups. Differences were considered statistically significant when P < 0.05.

### Data availability

The datasets generated during and/or analysed during the current study are available from the corresponding author on reasonable request.

## Electronic supplementary material

supplementary information
