# Supplementary material for: Temporal depolarization of mitochondria during M phase
Source: Sci Rep. 2017 Nov 22;7:16044. doi: 10.1038/s41598-017-15907-3 (PMC5700041; doi:10.1038/s41598-017-15907-3)
Supplement: Supplementary file 1 — supplementary information [file 41598_2017_15907_MOESM1_ESM.doc]

**Supplementary Information**

**Temporal depolarization of mitochondria during M phase**

Kotoe Hirusaki, Kaho Yokoyama, Kyunghak Cho, and Yoshihiro Ohta


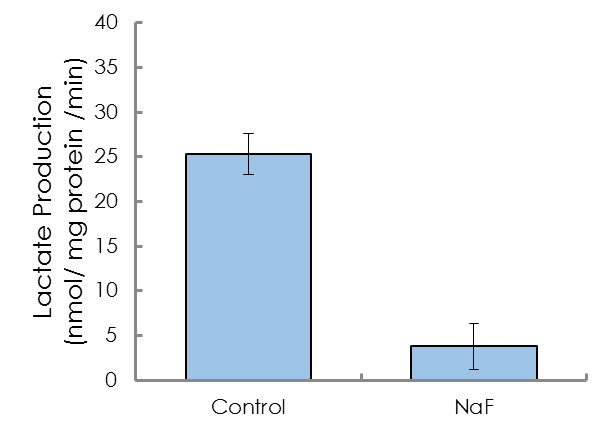


**Fig. S1 Effect of NaF on lactate production.** To evaluate lactate, cells were incubated for 15 min at 37 °C in DMEM without FBS. When NaF was added, the cells were pre-incubated for 10 min in the presence of 5 mM NaF, and NaF was present for the 15-min incubation period. N = 5. *P < 0.05.


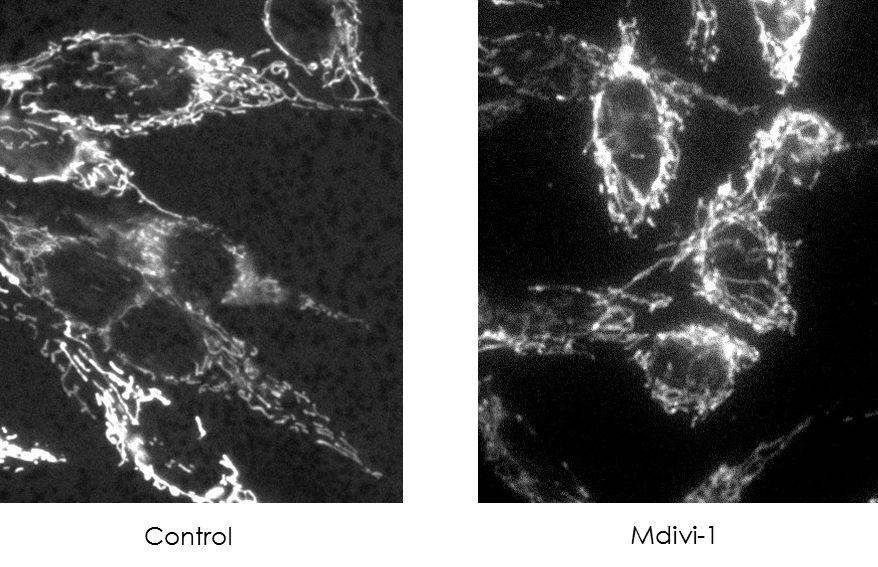


**Fig. S2 Effects of mdivi-1 on mitochondrial morphology.** Mitochondria in C6 cells were stained with 50 nM TMRE with/without mdivi-1. Bar, 5mm. When mdivi-1 was present, cells were incubated with 20 μM mdivi-1 for 6h at 37°C in the CO_2_ incubator prior to the measurements.
